# Healthcare Professionals and Unregulated Commercial Surrogacy in China: Ethical and Legal Challenges

**DOI:** 10.1007/s11673-025-10451-6

**Published:** 2025-04-14

**Authors:** Y. Luo, Y. Zhang

**Affiliations:** 1 University of Melbourne, Parkville, Victoria 3010 Australia; 2 Guangdong University of Foreign Studies, 2 Baiyun Avenue North, Guangzhou, China

**Keywords:** Commercial surrogacy, Healthcare ethics, Modern slavery, Exploitation, China, Bioethics

## Abstract

Recent reports from 2023 and 2024 have brought to light alarming instances of healthcare professionals in China being involved in commercial surrogacy arrangements. These cases reveal the dual roles these professionals play: active facilitation, which includes actions such as selling fraudulent birth certificates that contribute to baby trafficking, and passive involvement, where routine medical care is provided to surrogate mothers without full awareness of the surrogacy context. Current regulations in China broadly prohibit surrogacy-related medical activities but fail to differentiate between healthcare professionals who actively facilitate surrogacy arrangements and those who fulfil their professional obligations to patients. Drawing on professional ethics outlined in the revised 2021 Medical Practitioners Law of the People’s Republic of China and recent developments in modern slavery scholarship that emphasize the critical role of healthcare professionals in identifying and preventing exploitation, this paper argues that China has an opportunity to clarify and broaden the role of healthcare professionals in the context of surrogacy. Healthcare professionals should not be prohibited from providing medical care to surrogate mothers, provided they are not actively facilitating surrogacy arrangements. Simultaneously, they should be empowered to act as gatekeepers against exploitation. This would be an important step toward enhancing the current regulatory framework, ensuring better protections for children and women involved in surrogacy.

## Introduction

Commercial surrogacy in China has expanded into a substantial industry despite persistent concerns about the commodification of children and the exploitation of vulnerable individuals (Xiao et al. 2020). The absence of comprehensive regulation has cultivated a largely unregulated environment, where surrogacy practices—often facilitated through underground networks—are fraught with significant risks to the well-being of involved parties (Luo 2024). In this legal and ethical grey zone, healthcare professionals occupy a pivotal yet deeply contested role—a dimension that remains underexplored in scholarly discourse.

Recent reports have shed light on alarming cases in which healthcare professionals have participated in commercial surrogacy arrangements (Global Times 2023; Reuters 2024). These instances include the use of forged documentation and the sale of birth certificates, contributing to the formation of “baby-trafficking rings.” Such activities demonstrate how healthcare professionals can become integral to the functioning of unregulated surrogacy networks, blurring the boundaries between medical care and unlawful facilitation.

Beyond active facilitation, healthcare professionals may also find themselves passively involved in surrogacy arrangements. For example, an obstetrician might unknowingly provide prenatal care to a pregnant woman only to later learn that she is acting as a surrogate for another couple. However, existing regulations, such as the *Administrative Measures on Assisted Human Reproductive Technologies* (2001) and the *Ethical Principles of Human Assisted Reproductive Technology and Human Sperm Banks* (2003), broadly prohibit medical personnel from participating in any form of surrogacy (Luo 2024). These regulations fail to distinguish between healthcare professionals actively facilitating surrogacy arrangements and those simply fulfilling their duty of care to patients. This regulatory gap leaves healthcare professionals navigating a precarious ethical and legal landscape, where their efforts to prioritize patient welfare could inadvertently be construed as participation in prohibited practices.

This article first provides an overview of commercial surrogacy in China and delves into the vulnerabilities faced by surrogate mothers, who are often marginalized in both policy and practice. It then examines the active and passive involvement of healthcare professionals in surrogacy practices, highlighting the ethical dilemmas, legal challenges, and the pressing need for clear professional guidelines. The third part of this paper argues for a more nuanced regulatory approach informed by the professional ethics outlined in the revised *2021 Medical Practitioners Law of the People’s Republic of China* and recent developments in modern slavery scholarship (Hewitt and Nelson 2024). This paper argues that China has an opportunity to clarify and broaden the role of healthcare professionals in the context of surrogacy. Healthcare professionals should not be prohibited from providing medical care to surrogate mothers, provided they are not actively facilitating surrogacy arrangements. Simultaneously, they should be empowered to act as gatekeepers against exploitation. This would be an important step toward enhancing the current regulatory framework, ensuring better protections for children and women involved in surrogacy.

This paper aims to address the gap in scholarly discourse on the role of healthcare professionals in the context of unregulated surrogacy, emphasizing their potential to act as both protectors of vulnerable parties and enforcers of ethical and legal norms in reproductive healthcare. While this paper focuses on the situation in China, the ethical and legal challenges it addresses have broader implications for other countries where surrogacy is prohibited but continues to occur clandestinely.

## Commercial Surrogacy in China and the Insufficiency of Legal Protections

Commercial surrogacy in China exists in a legal grey area, neither explicitly prohibited nor officially sanctioned. This regulatory ambiguity has allowed surrogacy practices to operate largely underground, often exposing surrogate mothers to significant risks (Luo 2024). Surrogate mothers may encounter maternity-specific vulnerabilities, such as the physical and psychological strain of pregnancy and childbirth, insufficient access to quality medical care, and inadequate prenatal and postnatal support. These challenges are further intensified by the decision-making dynamics of intended parents, who may fail to fully recognize surrogate mothers as autonomous individuals with their own rights and needs. Driven by a deep desire to have a child, intended parents may, consciously or unconsciously, prioritize their own goals over the well-being of the surrogate mother.

China has established certain ethical guidelines and legal measures governing assisted reproductive technologies—such as the *Measures for the Administration of Assisted Human Reproductive Technology* (2001) and the *Ethical Principles of Human Assisted Reproductive Technology and Human Sperm Banks* (2003). However, the laws primarily focus on restricting medical institutions and professionals from participating in any form of surrogacy, yet they fail to offer a robust framework to address exploitation or advocate for the rights of surrogate mothers (Luo 2024). This legal silence leaves surrogate mothers in a precarious and vulnerable position, lacking formal protections against exploitation and with limited avenues for seeking redress. Furthermore, while these provisions unequivocally prohibit the involvement of medical professionals and healthcare institutions in surrogacy practices, they notably do not extend this prohibition to private individuals or non-medical entities acting as brokers (Luo 2024). The regulatory gap has facilitated the emergence of a substantial underground surrogacy market within China, operating in a legal grey area devoid of explicit statutory prohibition or endorsement (Luo 2024; Xiao et al. 2020).

## Healthcare Professionals’ Involvement and Ethical and Legal Challenges in Surrogacy

In this unregulated environment, some medical professionals and institutions clandestinely collaborate with surrogacy brokers to engage in surrogacy-related services (Global Times 2023; Reuters 2024). This active participation involves healthcare professionals directly engaging in the surrogacy process, such as coordinating arrangements, providing fertility treatments specifically for commercial surrogacy, or facilitating commercial surrogacy agreements. For instance, in May 2024, China’s National Health Commission initiated an investigation into a hospital in Chongqing following allegations of collaboration with commercial surrogacy agencies (Reuters 2024). Reports indicated that surrogate mothers used forged identification to give birth at the facility and the hospital issued fraudulent birth certificates for the newborns (Reuters 2024). Similarly, in November 2023, authorities in Wuhan suspended a hospital after it was implicated in commercial surrogacy operations and issuing fraudulent paternity test results (Global Times 2023). These fraudulent documents enabled intended parents to obtain birth certificates listing them as the biological parents, despite not being genetically related to the child (Global Times 2023). These hospitals are suspected of providing illicit assistance to commercial surrogacy arrangements and engaging in the buying and selling of infants.

However, a closer look at commercial surrogacy arrangements reveals scenarios where healthcare professionals may find themselves passively involved.^1^ For instance, imagine an obstetrician providing prenatal care to a pregnant woman who later discloses that she is carrying a child as a surrogate for another couple. In another scenario, during a routine examination, the obstetrician might recognize from medical records or other information that the pregnancy involves surrogacy. Such cases bring forward important ethical and legal considerations: What constitutes permissible care in these situations, and how can healthcare professionals navigate these boundaries without violating regulations? Additionally, how can they effectively document and justify their actions to prioritize patient welfare while safeguarding themselves from potential legal liability?

## The Need for Developing Professional Guidelines and the Opportunity to Strengthen Protection

Current regulations in China broadly prohibit surrogacy-related medical activities but fail to differentiate between healthcare professionals who actively facilitate surrogacy arrangements and those who fulfil their professional obligations to patients. This absence of clear directives leaves healthcare professionals uncertain about how to fulfil their obligations to ensure patient health without inadvertently contravening legal restrictions on surrogacy. When healthcare professionals do not actively engage in or promote surrogacy, providing medical care to surrogate mothers should not be considered a violation of professional ethics or legal regulations. Healthcare providers are fundamentally entrusted with the responsibility to safeguard the health and well-being of their patients. The *Medical Practitioners Law of the People’s Republic of China*, revised in August 2021,^2^ emphasizes professional ethics by requiring practitioners to “respect life,” “heal the wounded,” and “maintain boundless love.” These principles establish a duty for healthcare professionals to provide compassionate care and support to all patients, including those involved in controversial or unregulated practices like commercial surrogacy. Professional ethics such as “respect life” create a strong obligation for healthcare professionals to intervene and provide care that minimizes harm. These principles encourage them to regard surrogate mothers not as participants in an ethically complex arrangement but as patients deserving of compassionate, high-quality care.

A robust guideline is therefore essential to provide clear directives that enable healthcare professionals to navigate the complexities of surrogacy-related care while upholding their ethical obligations and complying with legal restrictions. Such guidelines should distinguish between providing essential medical care to protect the health and safety of surrogate mothers and actively facilitating surrogacy arrangements, which contravene legal and ethical standards. To effectively make this distinction, several key criteria can be applied. For example, the knowledge of the surrogacy status should play a crucial role. If a healthcare professional is unaware that a patient is a surrogate mother and provides standard medical care without any additional involvement, this may constitute unknowing or passive involvement. However, if the healthcare professional is aware of the patient’s surrogacy status and engages in actions beyond standard medical care to support or facilitate the arrangement, this may shift their role to active facilitation.

Intent and purpose should further distinguish the nature of involvement. When the actions of healthcare professionals are solely aimed at providing medical care and improving the patient’s health without ulterior motives related to surrogacy, their involvement should be considered passive. Conversely, if their actions are intended to support, coordinate, or enable surrogacy arrangements, often involving additional non-medical services, this can indicate active facilitation. This could include activities such as coordinating surrogacy agreements, liaising with surrogacy agencies, falsifying medical records or birth certificates, and providing medical services specifically intended to support illegal surrogacy practices. For instance, performing procedures solely for the purpose of implanting embryos as part of a commercial surrogacy arrangement would fall outside the permissible scope of care under such guidelines. Financial transactions also can play a pivotal role in distinguishing involvement levels. Providing care without any financial transactions related to surrogacy should align with passive involvement, whereas receiving or providing financial incentives to support surrogacy arrangements should clearly mark active facilitation.

After establishing clear guidelines based on these criteria, the framework could specify that healthcare professionals are permitted to provide essential medical services—such as prenatal and postnatal care, routine check-ups, and treatment for pregnancy-related complications—when they are passively involved in surrogacy procedures. This can ensure the health and safety of surrogate mothers and aligns with their professional duty of care. Conversely, the guidelines should explicitly prohibit healthcare professionals from actively participating in or facilitating surrogacy arrangements.

Recent developments in modern slavery scholarship underscore the pivotal role healthcare professionals play in identifying and mitigating exploitation, particularly in cases of human trafficking. Hewitt and Nelson (2024) highlight that under the UK’s *Modern Slavery Act* (2015), modern slavery encompasses offenses such as slavery, servitude, forced or compulsory labour, and human trafficking. They advocate for comprehensive training programmes to equip healthcare workers with the skills to recognize signs of exploitation and provide appropriate care, ensuring victims receive necessary support and protection. In the United States, where medical practice is largely regulated by individual states, healthcare professionals are bound by mandatory reporting requirements for the suspected abuse or neglect of minors, as well as for the exploitation, abuse, or self-neglect of elderly or vulnerable adults (Geiderman and Marco 2020). These laws underscore the critical responsibility of healthcare workers to safeguard vulnerable individuals and uphold public welfare by reporting exploitation to authorities.

By adopting similar strategies, China has the opportunity to strengthen its regulatory framework and better protect women and children involved in surrogacy arrangements. In the context of surrogacy, lessons from modern slavery legislation can guide the development of protocols that empower healthcare professionals to recognize and address signs of exploitation effectively. Drawing from their unique position as frontline caregivers, healthcare workers have access to surrogate mothers throughout pregnancy and postpartum periods, enabling them to monitor both physical and emotional well-being. This privileged access can equip healthcare professionals to detect early indicators of exploitation, coercion, or health risks inherent in unregulated surrogacy practices. For instance, during routine medical consultations or examinations, healthcare professionals can identify unusual patterns in medical histories, such as multiple pregnancies without accompanying children or husbands, and minimal recovery time between pregnancies. Such patterns may indicate that a woman is involved in surrogacy and could be at significant health risk due to the physical and emotional demands placed on her. These irregularities can provide critical opportunities for healthcare providers to intervene and address potential exploitation. By educating patients about the health risks of surrogacy, healthcare professionals can empower women to make informed decisions about their reproductive health. Moreover, they can offer confidential support and referrals to social services or counselling centres, ensuring that vulnerable women receive the necessary assistance without feeling coerced. Through these measures, healthcare professionals can effectively act as a crucial barrier against the exploitation occurred in surrogacy practices, safeguarding the health and rights of women.

Moreover, mandatory reporting requirements, as observed in the obligations of healthcare professionals in the United States, can be adapted to the surrogacy context in China. Healthcare professionals should be required to report surrogacy cases to a regulatory or supervisory body, such as a hospital ethics committee or a governmental agency, thereby creating an official record of their involvement. This dual approach ensures that healthcare providers fulfil their professional obligations without appearing complicit in unregulated commercial surrogacy. By doing so, healthcare professionals can demonstrate that their actions are driven by ethical and medical concerns rather than financial profit or support for surrogacy arrangements. This reporting mechanism acts as a safeguard, signalling that they are not engaging in or endorsing the practice but are prioritizing the health needs of individuals who may be at risk of exploitation.

While mandatory reporting laws are designed to protect vulnerable populations, they prioritize the welfare of the patient and society (non-maleficence and beneficence) over patient autonomy and confidentiality (Geiderman and Marco 2020). Reporting without the patient’s consent may cause distrust or discourage individuals from seeking care. Failing to report, however, could allow harm to continue unchecked. Moreover, reporting encounters with surrogate mothers to authorities may lead to unintended negative consequences. While surrogate mothers may not be subject to legal punishment, reporting could expose them to police investigations, legal scrutiny, social stigma, or other forms of harm. Therefore, establishing reporting mechanisms specifically designed to protect surrogate mothers is essential. Such protocols should outline when and how to report, to whom the report should be directed, and what information should be included, ensuring that reporting is conducted appropriately and sensitively. For example, healthcare providers could contribute to anonymized data collection that informs authorities about the prevalence of unregulated surrogacy without revealing specific patient identities. Adopting models such as safe-harbour laws, enacted in several U.S. states, could further enhance protections (Geiderman and Marco 2020). These laws recognize individuals involved in illegal activities, like human trafficking, as victims in need of healthcare and support services rather than treating them as criminals (Geiderman and Marco 2020). Implementing similar policies in China could protect surrogate mothers from negative repercussions when seeking medical care.

Building on suggestions by Hewitt and Nelson (2024), there is a pressing need for the development of guidelines and training programmes in China that equip healthcare professionals with the necessary skills and knowledge to identify and respond appropriately to exploitation in commercial surrogacy. Victims of modern slavery often do not perceive themselves as victims and may be reluctant to disclose their circumstances due to fear of reprisal, mistrust of authorities, or manipulated narratives provided by their traffickers (Hewitt and Nelson 2024). Similarly, surrogate mothers in exploitative arrangements may feel trapped, either financially or emotionally, and hesitate to reveal their circumstances. Healthcare professionals must develop a heightened awareness of the common indicators of exploitation, including physical, psychological, and systemic signs. Surrogate mothers, may fear judgment or repercussions and require a safe environment to disclose their experiences. Building trust through private, empathetic communication is also important.

One area of urgent concern is the potential overlap between unregulated surrogacy and baby trafficking. Signs that healthcare professionals should be attuned to include discrepancies in birth documentation, such as forged or altered birth certificates, inconsistent parental identification, or vague explanations regarding the custody of the child. Additionally, surrogate mothers presenting with little to no information about the intended parents, lack of access to their own identification documents, or signs of coercion by intermediaries may signal exploitative practices that could involve trafficking. Healthcare professionals should also be alert to situations where newborns are immediately separated from their birth mothers without clear legal procedures or documentation.

## Conclusion

This paper serves as a starting point to explore the complex and underexamined role of healthcare professionals in unregulated surrogacy arrangements in China. Drawing lessons from modern slavery practices, it highlights the need for greater awareness, training, and systemic reforms to address the vulnerabilities and potential exploitation associated with unregulated surrogacy practice. While this discussion identifies key areas of concern and offers initial strategies for safeguarding and ethical care, it merely scratches the surface of a multifaceted issue. Future research and dialogue are needed to develop comprehensive guidelines that balance the ethical obligations of healthcare professionals with the rights and well-being of all individuals involved in surrogacy arrangements.

China now faces a timely opportunity to reconsider the scope of the role healthcare professionals play in surrogacy. Developing clear professional guidelines is essential to resolve the ethical and legal dilemmas practitioners encounter. These guidelines should delineate the boundaries between permissible medical care and prohibited participation and facilitation of surrogacy arrangements, thereby empowering healthcare providers to deliver compassionate, high-quality care to surrogate mothers while upholding ethical and legal standards. By drawing on lessons from modern slavery frameworks, these measures can position healthcare professionals as gatekeepers against exploitation. A reconsidered and well-defined role for healthcare professionals can strengthen the regulatory framework, ensuring enhanced protections for women and children involved in surrogacy.

## Footnotes

1 According to the news (Guosheng 2024), when the surrogate mother arrived at the hospital, the doctor initially treated her without any knowledge that the pregnancy was part of a surrogacy arrangement. After the surrogate successfully delivered the baby, the intermediary (or agent) revealed the truth, admitting that the woman was involved in a surrogacy arrangement.

2 Medical Practitioners Law of the People’s Republic of China. (Adopted at the 30 th Meeting of the Standing Committee of the Thirteenth National People’s Congress of the People’s Republic of China on August 20, 2021). **Article 3**: Medical practitioners shall put the people and life first for the humanitarian spirit and promote the lofty professional spirit of respecting life, healing the wounded and the sick, being willing to dedicate themselves and maintaining boundless love. They shall observe professional ethics and the code of practice, improve practicing skills and perform the sacred duties of preventing and treating diseases and protecting people’s health.
